# Joint image reconstruction and segmentation of real-time cardiovascular magnetic resonance imaging in free-breathing using a model based on disentangled representation learning

**DOI:** 10.1016/j.jocmr.2025.101844

**Published:** 2025-01-24

**Authors:** Tobias Wech, Oliver Schad, Simon Sauer, Jonas Kleineisel, Nils Petri, Peter Nordbeck, Thorsten A. Bley, Bettina Baeßler, Bernhard Petritsch, Julius F. Heidenreich

**Affiliations:** a Department of Diagnostic and Interventional Radiology, University Hospital Würzburg, Würzburg, Germany; b Comprehensive Heart Failure Center Würzburg, Würzburg, Germany; c Department of Internal Medicine I, University Hospital Würzburg, Würzburg, Germany

**Keywords:** Magnetic resonance imaging, Disentangled representation learning, Semantic segmentation, Cardiac function, Machine learning, Deep learning

## Abstract

**Background:**

To investigate image quality and agreement of derived cardiac function parameters in a novel joint image reconstruction and segmentation approach based on disentangled representation learning, enabling real-time cardiac cine imaging during free-breathing.

**Methods:**

A multi-tasking neural network architecture, incorporating disentangled representation learning, was trained using simulated examinations based on data from a public repository along with cardiovascular magnetic resonance (CMR) scans specifically acquired for model development. An exploratory feasibility study evaluated the method on undersampled real-time acquisitions using an in-house developed spiral balanced steady-state free precession pulse sequence in eight healthy participants and five patients with intermittent atrial fibrillation. Images and predicted left ventricle segmentations were compared to the reference standard of electrocardiography (ECG)-gated segmented Cartesian cine with repeated breath-holds and corresponding manual segmentation.

**Results:**

On a 5-point Likert scale, image quality of the real-time breath-hold approach and Cartesian cine was comparable in healthy participants (RT-BH: 1.99 ± 0.98, Cartesian: 1.94 ± 0.86, p = 0.052), but slightly inferior in free-breathing (RT-FB: 2.40 ± 0.98, p < 0.001). In patients with arrhythmia, both real-time approaches demonstrated favorable image quality (RT-BH: 2.10 ± 1.28, p < 0.001, RT-FB: 2.40 ± 1.13, p < 0.01, Cartesian: 2.68 ± 1.13). Intra-observer reliability was good (intraclass correlation coefficient = 0.77, 95% confidence interval [0.75, 0.79], p < 0.001). In functional analysis, a positive bias was observed for ejection fractions derived from the proposed model compared to the clinical reference standard (RT-BH mean: 58.5 ± 5.6%, bias: +3.47%, 95% confidence interval [−0.86, 7.79%], RT-FB mean: 57.9 ± 10.6%, bias: +1.45%, [−3.02, 5.91%], Cartesian mean: 54.9 ± 6.7%).

**Conclusion:**

The introduced real-time CMR imaging technique enables high-quality cardiac cine data acquisitions in 1–2 min, eliminating the need for ECG gating and breath-holds. This approach offers a promising alternative to the current clinical practice of segmented acquisition, with shorter scan times, improved patient comfort, and increased robustness to arrhythmia and patient non-compliance.

## Graphical abstract

**Figure:**
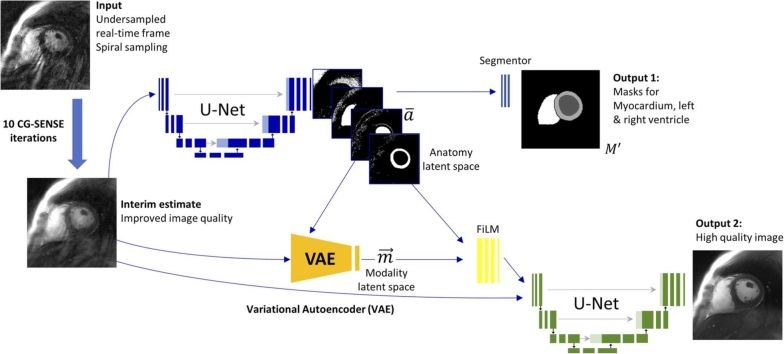

## 1 Introduction

Dynamic cine cardiovascular magnetic resonance (CMR) imaging is fundamental for functional assessment in cardiac magnetic resonance imaging (MRI), regardless of underlying pathology. In clinical routine, electrocardiography (ECG)-gated segmented sampling with Cartesian readouts remains the method of choice at most centers, providing time-resolved views with sufficient spatial and temporal resolution as well as high signal-to-noise ratio (SNR). To shorten breath-holds required acquiring approximately 15 spatiotemporal views (2D + *t*) that cover the heart from base to apex, a slight acceleration (R ∼ 2–3) is typically achieved using parallel imaging. Segmentation of the different compartments of the heart (myocardium, left ventricle [LV], and right ventricle [RV]) is subsequently performed during end-systolic and end-diastolic phases, to derive functional parameters such as stroke volume and ejection fraction (EF). While these tasks were done manually for a long time, semi-automatic or fully automatic tools [1] are increasingly being used for improved efficiency.

Segmented cine imaging provides high-quality heart depictions when patients are compliant, i.e., they can hold their breath for a few seconds and have regular heartbeats with reliable ECG recognition. Often these requirements are not met and severe motion artifacts can corrupt the resulting images. This susceptibility arises from the method’s large temporal footprint, which typically spans multiple cardiac cycles. Efforts have been underway for years to establish alternative techniques for robust, real-time, free-breathing acquisitions [2]. Yet, these have not largely replaced traditional segmented cine imaging and remain fallback options for challenging cases. Additionally, the necessary acceleration often leads to a loss in quality that radiologists are generally reluctant to accept.

Various strategies have been developed to reduce acquisition times for real-time cardiac MRI, targeting different parts of the imaging chain. Readout trajectories, such as echo planar imaging or spirals, which are per se more efficient than Cartesian sampling, have been exploited for rapid data sampling [3], [4]. However, image distortions due to off-resonance and gradient imperfections probably can pose significant challenges. In recent years, these issues have been addressed effectively by correction methods based on a gradient response function [5], [6], [7]. Acquisitions that violate the Nyquist criterion (referred to as “undersampling”) became popular especially with the advent of parallel imaging [8], allowing for ECG-free, free-breathing imaging [9]. The introduction of compressed sensing (CS) to MRI [10] enabled reconstructions of real-time measurements with temporal and spatial resolution comparable to segmented cine techniques [11]. These “model-based” approaches have also been combined with non-Cartesian trajectories to leverage multiple acceleration potentials simultaneously [7], [12], [13]. Finally, data-driven methods, i.e., machine learning (ML)-based reconstruction techniques, are increasingly being trained and applied to convert undersampled acquisition into images of higher quality [14]. Many of these ML approaches can deliver real-time cardiac images with much lower latency than traditional CS models, which frequently require multiple iterations of associated optimization algorithms. This lower latency is not only desirable for standard volumetric assessment but essential for advanced applications, such as interventional cardiac MRI [15].

To enhance the efficiency and stability of MR-based cardiac volumetry, we propose a new technique for high-quality, real-time dynamic cine imaging with fast image reconstruction and postprocessing. Data sampling is based on balanced steady-state free precession (bSSFP) and undersampled spiral trajectories, corrected by a gradient system transfer function (GSTF) [7]. Acquisitions are reconstructed and segmented in a joint fashion using a novel multi-tasking neural network exploiting disentangled representation learning. The model was trained on simulated data derived from an open database as well as data acquired specifically for this project. An exploratory feasibility study then evaluated the method in healthy volunteers and patients with intermittent atrial fibrillation.

We published a conference abstract introducing a preliminary version of the approach based on radial data from an open database [16]. For the cited work, segmented cine acquisitions in breath-hold were retrospectively undersampled to simulate acceleration. We now have trained and extensively validated the method with more efficient spiral (real-time) data, which were prospectively acquired for this purpose both under breath-hold and free-breathing.

## 2 Methods

### 2.1 Study population and ethics license

This prospective single-center study was approved by the local ethics committee (license ID 173/22_skpm). Written informed consent was obtained from all participants. CMR was acquired in 16 healthy study participants and 5 patients with cardiac arrhythmia. Data from eight healthy participants were used for training, while data from the remaining eight as well as from all five patients were used to evaluate the method. Inclusion criteria were age >18 years and for patients the presence of intermittent atrial fibrillation. The presence of arrhythmia was confirmed by ECG during the MRI. Exclusion criteria were any common contraindication to MRI.

### 2.2 MR pulse sequence and gradient waveform correction

**Fig. 1:**
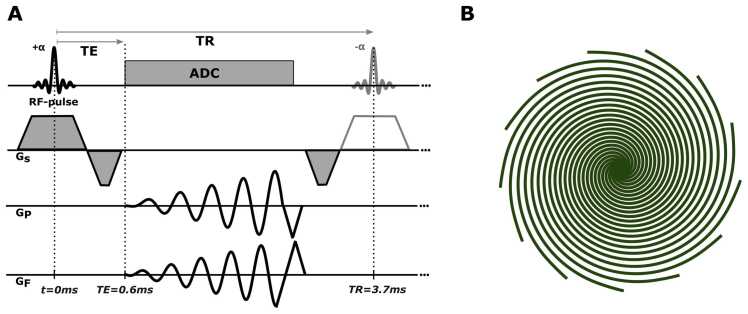
(A) Spiral balanced steady-state free precession MR pulse sequence. (B) Thirteen consecutive spiral arms, equally distributed across 2π, represent the k-space trajectory for one real-time frame (48 ms). To acquire training data for xSDNet in healthy volunteers, this pattern was repeated for a duration exceeding one RR interval. Subsequently, the pattern was rotated to fill the largest gap in k-space. If data are acquired in breath-hold, and a sufficient number of orientations is sampled (>5 in this case), both real-time and segmented k-spaces (self-gating with DC signal) can be determined from the same acquisition. *MR* magnetic resonance, *xSDNet* extended spatial decomposition network, *RF* radio frequency, *RR* interval between successive R peaks, *TE* echo time, *TR* repetition time, *ADC* analog-to-digital converter, *DC* direct current.

A spiral bSSFP pulse sequence (Fig. 1 and Table 1) was implemented on a 1.5T whole-body MR system (Siemens Magnetom Avanto Fit, Siemens Healthcare GmbH, Germany). A single real-time frame was acquired using 13 consecutive arms (repetition time = 3.7 ms), which were equally distributed in k-space and feature a temporal footprint of 48 ms. The undersampling factor with respect to Nyquist sampling was about 5 in the center of k-space and increased toward 15 in the periphery. An radio frequency phase alternation of 180° (±α) was fixed throughout all measurements and no frequency scout was acquired in addition to the shimming of the cardiac region of interest. Triangular rewinders were appended to the spiral readout to null the zeroth gradient moment.

**Table 1:** Acquisition parameters of the fully sampled segmented Cartesian bSSFP cine as well as the undersampled spiral real-time bSSFP pulse sequence.

Sequence parameter	Segmented Cartesian cine bSSFP	Undersampled spiral real-time bSSFP
Slice thickness (mm)	8	8
Flip angle (°)	70	70
Spatial resolution (mm)	1.25 × 1.25	1.29 × 1.29
FOV (mm)	320 × 260	592 × 592
Image matrix (px)	256 × 208	512 × 512
TR (ms)	3.94	3.70
TE (ms)	1.97	0.61
Temporal resolution (ms)	44±4	48
Pixel bandwidth (Hz/pixel)	528	407
GRAPPA	2	-

For the latter, the spatial resolution corresponds to k_max_ (highest k-value sampled in k-space) of the spiral trajectory. The FOV represents the reconstructed FOV.

*bSSFP* balanced steady-state free precession, *TE* echo time, *TR* repetition time, *GRAPPA* generalized autocalibrating partially parallel acquisition, *FOV* field of view.

All parameters are given in the units shown in brackets on the left. ± indicates the standard deviation when parameters varied between measurements. For the spiral acquisition, the spatial resolution corresponds to k_max (highest k-value in k-space) of the undersampled trajectory. The FOV represents the reconstructed FOV.

**Fig. 2:**
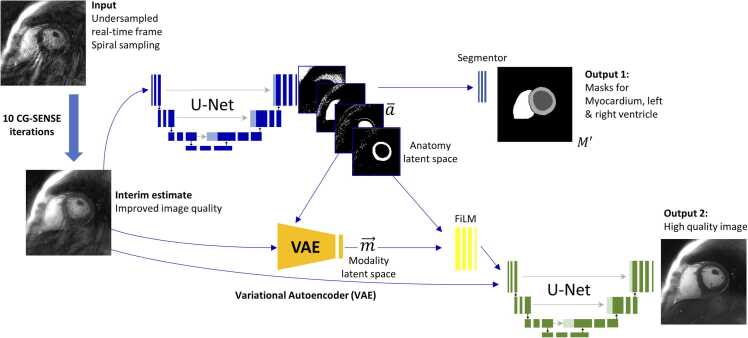
Illustration of the proposed xSDNet to provide joint reconstruction and segmentation of undersampled cardiac real-time frames. *xSDNet* extended spatial decomposition network

To train the proposed artificial neural network, slightly altered acquisitions were additionally performed in healthy study participants: Data were sampled for the duration of a breath-hold of approximately 10 s for each of the 10–15 short-axis slices. The trajectory patterns were rotated after being repeated for a period exceeding one RR interval. By acquiring eight different orientations within one breath-hold, both the binning of real-time frames and the determination of fully sampled k-spaces—segmented across multiple RR intervals based on self-gating—were possible. This approach delivered matching pairs of undersampled and fully sampled data of the same cardiac phase, which were ultimately used to train the model described in Section 2.4 (refer to Supplemental Material Section 1, for more details on the acquisition patterns). Non-uniform fast Fourier transform (NUFFT) reconstructions of the former (undersampled real-time) represent the input of the proposed method (see “Input” in Fig. 2), NUFFT reconstructions of the latter (segmented cine), complemented by a manual segmentation thereof represent the two output images (see “Output 1” and “Output 2” in Fig. 2). All acquisitions with spiral gradient waveforms were corrected using a GSTF [17] in post-correction, i.e., the GSTF was determined once for the system used, and was subsequently applied to obtain the k-space-trajectory actually played out by the scanner. This corrected trajectory was then used for all reconstruction throughout this study.

### 2.3 Disentanglement

Disentangled representation learning (DRL, [18]) aims at factorizing data into several meaningful and disjoint (and thus independently varying) features. Compared to “black box” models, this more “human-like approach” to model data, potentially features increased interpretability, versatility, improved generalization and application robustness as well as multi-tasking capabilities. For medical imaging, Chartsias et al. [19] proposed a spatial decomposition network (SDNet) to disentangle radiologic images of the heart into an anatomical latent space and a modality latent space encoding contrast information. This approach enabled automatic image segmentation and style transfer of cardiac MRIs, as two example applications of this intuitive and general model. Here, we describe a network of similar nature that simultaneously reconstructs and segments undersampled functional MR “real-time” images of the heart.

### 2.4 Model for joint reconstruction and segmentation

The proposed joint reconstruction and postprocessing strategy for cardiac real-time MRI is illustrated in Fig. 2. The input is represented by the undersampled (spiral) raw data acquired for a single two-dimensional (2D) real-time frame (48 ms footprint, Section 2.2). These data are first subjected to 10 iterations of a CG-SENSE [20] algorithm, incorporating graphics processing unit (GPU)-based convolution gridding [21] for fast runtimes. By exploiting the encoding capacity of the employed phased-array coil, this leads to an interim estimate of the real-time frame which already features less undersampling artifacts and improved image quality with respect to the naïve gridding reconstruction of the measured data. A temporal average image across multiple time frames was used to determine coil sensitivities for this purpose [22].

The obtained interim 2D image is subsequently processed by a multi-compartment neural network which we refer to as extended spatial decomposition network (xSDNet) in the following. The architecture is derived from the SDNet model proposed in [19] and was customized to both reconstruct and segment undersampled images with high quality. The upper path in Fig. 2 depicts the application of a U-Net [23] to determine an anatomical latent space, featuring eight anatomical factors represented by 2D Boolean matrices. This latent space builds the input to a segmentor, which is a shallow, fully convolutional neural network consisting of three convolutional layers with batch normalization and LeakyReLu activation in between, and a softmax classifier as final operator. The output is a mask for LV and RV as well as for myocardial tissue (Output 1).

The lower path in Fig. 2 supplies the interim image to a variational autoencoder to generate a “modality vector” of length eight, encoding the contrast information of the input. This modality latent space is then fused with the anatomy latent space from the upper path by a decoding convolutional network featuring feature-wise linear modulation-based normalization [24]. The resulting output was stacked with the interim estimate delivered by the CG-SENSE iterations to build the input of a final U-Net, which provides a high-quality reconstruction of the 2D real-time frame (Output 2). More detailed information on the architecture of the proposed model can be found in the Supplementary Material under Section 2.

### 2.5 Training

xSDNet was trained using a combination of simulated (set 1) and self-acquired data (set 2). The simulated data (set 1) were derived from a subset of 96,630 images from the “Kaggle Data Science Bowl Cardiac Challenge Data” [25], which were automatically segmented for LV, RV, and myocardium using Bai’s model [1]. Undersampled spiral raw data were additionally simulated for each image by superimposing artificial coil sensitivities (randomly varied for each sample) and applying a forward NUFFT operator [21], initialized by the spiral trajectory used for real-time imaging. Those data were subjected to 10 CG-SENSE iterations to obtain the input of xSDNet (interim estimate, Fig. 2). The two corresponding output labels are represented by the fully sampled image from the Kaggle dataset (Output 2) and the segmentation masks (Output 1). Set 1 offers a wide variety of heart shapes, which can be particularly useful for training the anatomy path of xSDNet (the upper part of Fig. 2).

In addition, dedicated training data were acquired in eight healthy participants (set 2). Ten to 15 short-axis slices were sampled in breath-hold as detailed in the second paragraph of Section 2.2 and the Supplementary Material Section 1. “Fully sampled” images were obtained by applying the inverse NUFFT operator to 104 (= 8 × 13) self-gated spiral arms from eight consecutive heartbeats (Output 2). These images were segmented manually for LV, RV, and myocardium by an MD candidate, who was trained for manually segmenting cardiac MR images by an expert (J.F.H) with 7 years of experience in cardiac MRI. All segmentations used for this study were reviewed by the expert. Obtained segmentations represent the label for Output 1. Finally, interim estimates of the real-time frames—matched to the cardiac phases of the self-gated frames—were obtained by subjecting the 13 equidistant spiral arms (with a 48-ms footprint) to 10 CG-SENSE iterations (input of xSDNet). Exactly 2010 input images with corresponding labels were obtained from the in-vivo study, which allow fitting the xSDNet model for the aimed target reconstruction and postprocessing task.

xSDNet was trained for 200 epochs. In each epoch, all data from set 2 were pooled with 282 randomly chosen samples from set 1, and, subsequently, a random horizontal and/or vertical flip combined with a random rotation by an angle ∈ [0°, 90°, 180°, 270°] was performed for data augmentation. While remaining losses were used as proposed in the original SDNet [19], a perceptual loss [26] was used for the reconstruction path in our approach.

### 2.6 In-vivo study

The proposed method was tested in undersampled real-time spiral cine MRI at 1.5T in eight healthy participants and five patients with intermittent atrial fibrillation. For better comparability with a clinical reference (see description below), data were first acquired in breath-hold. For patients, acquisition time was set to a breath-hold length of 4.5 s, resulting in 3–4 RR intervals per slice, depending on heart rates. For healthy volunteers, a prolonged breath-hold was used to preserve the possibility of determining a fully sampled (segmented) image series (additional analysis in Supplementary Material Sections 5 and 6).

Real-time data were acquired in free-breathing for the same slices in an end-to-end fashion. For each slice, data were sampled for 5.7 s. For a stack of 10–15 short-axis views, the entire scan time consequently corresponds to 57–86 s for full coverage of the LV. All data (breath-hold and free-breathing) were subjected to joint reconstruction and segmentation by xSDNet and will be referred to as “test data” in the following.

Corresponding segmented Cartesian bSSFP cine series with ECG gating were acquired in breath-hold as the current clinical reference standard (“reference data”), again for the same slices as for the real-time investigations (Table 1).

### 2.7 Comparison of disentanglement model with alternative methods

The test data reconstructed by xSDNet were compared to reconstructions by CG-SENSE, l1-wavelet-based CS [10] (implementation of BART toolbox, [27]), low-rank plus sparse (LRS, [28]), and a variational network [14]. It is important to note, that only LRS exploits redundancies in the temporal domain *t*, by applying a threshold to the series subsequent to a Fourier transform along *t* and an approximation of a corresponding spatiotemporal Casorati-matrix as low-rank. All other models reconstruct 2D frames individually and independently from other cardiac phases.

### 2.8 Expert reader study

Two expert readers (J.F.H. 7 years of experience in cardiac MRI, N.P. 3 years of experience) rated the real-time images as reconstructed by xSDNet and the corresponding ground truth images obtained by fully sampled segmented Cartesian acquisitions with ECG-triggering. Experts were blinded to the reconstruction technique and the following items were rated: artifacts in the blood pool, artifacts in the myocardium, sharpness of the endocardium, sharpness of the epicardium, depiction of dynamics, and overall image quality. The items were rated on a 5-point Likert scale following the definitions for image quality assessment with 1: excellent, 2: very good, 3: good without impact on diagnostic quality, 4: fair with impact on diagnostic quality, and 5: poor with strong impact on diagnostic quality.

### 2.9 Quantification of cardiac function

For comparison of cardiac function, endocardial and epicardial contours of the LV and contours of RV were segmented manually in Cartesian cine series (MEVISdraw v1.0, Fraunhofer MEVIS, Bremen, Germany). Real-time data in breath-hold and free-breathing were segmented fully automatically by xSDNet. Since several cardiac cycles were covered in real-time, median values were determined for the automatically determined local maxima and minima. Ejection fraction was calculated from end-systolic and end-diastolic volumes.

### 2.10 Statistical analysis

Statistical analysis was performed with Python 3.12 using Pingouin [29] and SciPy libraries. For the expert reader study, intraclass correlation coefficient (ICC) estimates and their 95% confidence intervals were calculated based on a mean rating (k = 3), consistency, and two-way mixed-effects model. Friedman’s test was used for comparison of xSDNet methods with Cartesian reference. Post-hoc pairwise comparison was performed with Wilcoxon ranked sum test. A p-value <0.05 was considered statistically significant.

## 3 Results

### 3.1 Comparison of disentanglement model with alternative methods

**Fig. 3:**
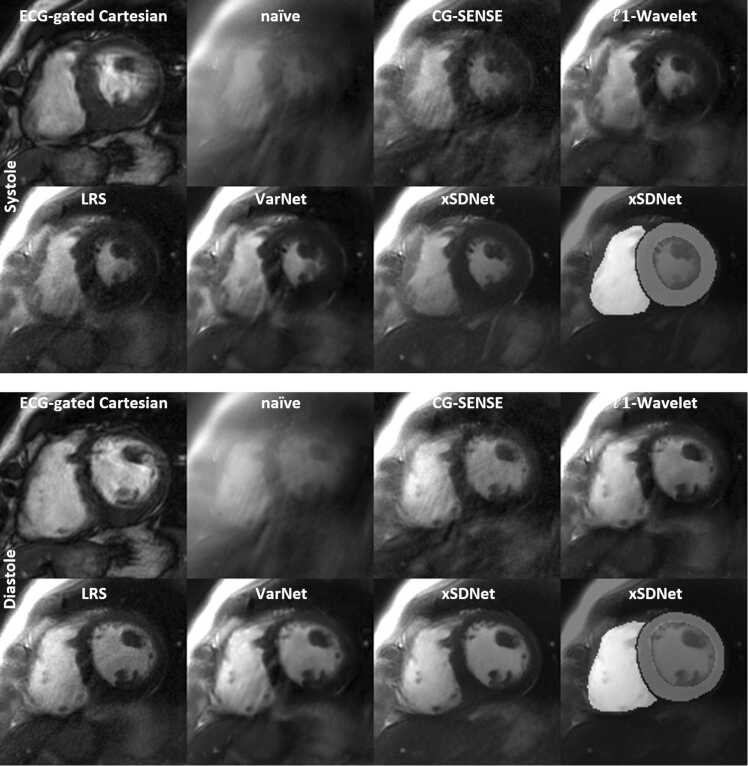
Performance of xSDNet in comparison to alternative reconstruction methods (breath-hold acquisitions) for a patient suffering from arrhythmia, both for a systolic and a diastolic cardiac phase. The image on top left shows the result from the clinical method based on ECG-gated and segmented Cartesian bSSFP, respectively. All other images were reconstructed from undersampled spiral real-time acquisitions with a temporal footprint of 48 ms. The final image shows the second output of xSDNet, i.e., a segmentation of the image, as overlay. See Supplementary Material for a dynamic view (supplementary_video_1.gif). *xSDNet* extended spatial decomposition network, *ECG* electrocardiography, *bSSFP* balanced steady-state free precession, *LRS* low-rank plus sparse

In Fig. 3, a systolic and a diastolic frame is depicted for the fully sampled ECG-gated Cartesian reference together with a “naïve” gridding reconstruction of undersampled spiral real-time acquisitions in breath-hold from a patient with atrial fibrillation. Real-time images were reconstructed using various methods as indicated (CG-SENSE, LRS, Variational Network, xSDNet). In the final image, segmentation masks provided by xSDNet are overlaid on the image output.

The Cartesian reference (ECG-gated) shows clear motion artifacts, in both the systolic and the diastolic frame. This indicates that ECG gating was not properly working for the irregular heartbeat, which corrupted the segmented acquisition and impaired the evaluation of cardiac dynamics. The naïve gridding reconstruction of the spiral real-time frames illustrates the severity of undersampling/aliasing artifacts that would degrade image quality if no advanced reconstruction technique were applied. In general, all images reconstructed from spiral data show increased blurring of fat in comparison to the Cartesian trajectory. CG-SENSE can significantly reduce the level of undersampling artifacts; however, residual streaks are apparent and SNR is sub-optimal. Using an additional l1-wavelet regularization further improved image quality, however, undersampling can still be recognized and typical wavelet artifacts (“blocky-effect”) can be seen in mild form. LRS features an overall sharper appearance, but slightly noisier as other techniques, except SENSE. The Variational Network resulted in clearly higher SNR, while residual artifacts are slightly higher than for LRS. Overall best image quality was observed for the proposed xSDNet model. Both systolic and diastolic view represent sharp depictions of the respective cardiac phase, with high SNR and lowest artifact level. Moreover, the model simultaneously delivers an accompanying segmentation, which can be seen as an overlay in the final image of the figure. In Supplementary Material, a complimentary dynamic view can be seen, showing all frames of Fig. 3 (supplementary_video_1.gif). Motion artifacts within the ECG-gated Cartesian cine are even more clearly recognizable with respect to the static images, which is largely avoided by the real-time series. xSDNet shows high spatial and temporal sharpness and low artifact level.

**Fig. 4:**
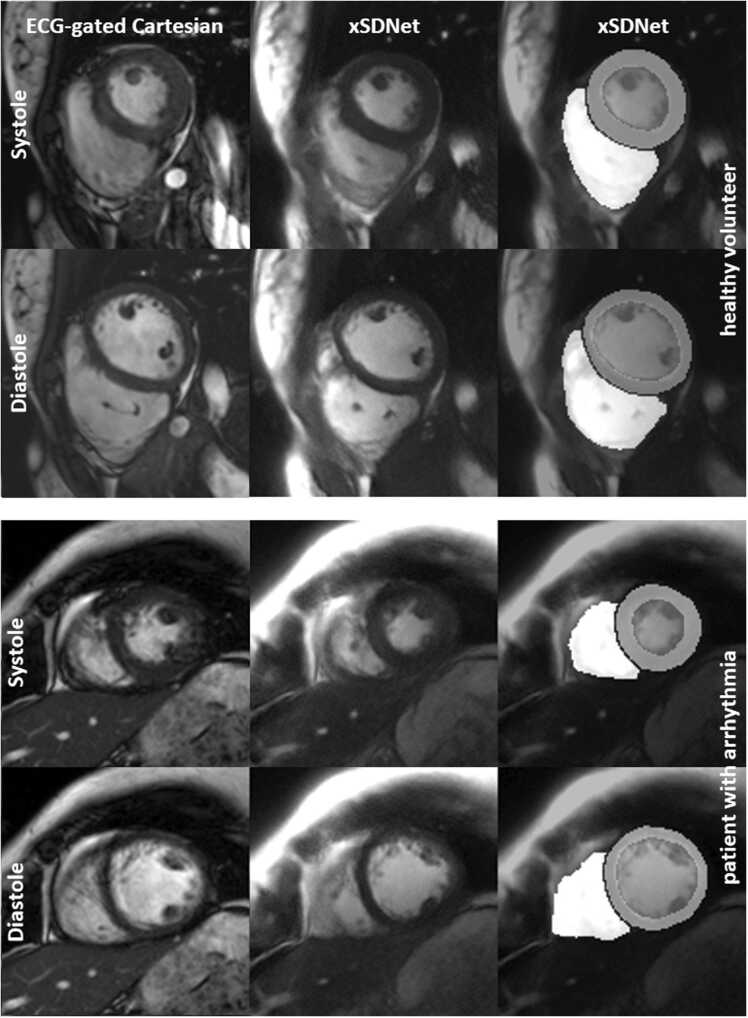
Performance of xSDNet-based reconstruction and segmentation of real-time acquisitions in free-breathing in comparison to ECG-gated Cartesian cine in breath-hold. Views differ slightly between xSDNet and cine due to breathing motion and significant delay between the two acquisitions. See Supplementary Material for dynamic views (supplementary_video_2.gif and supplementary_video_3.gif). *xSDNet* extended spatial decomposition network, *ECG* electrocardiography

Fig. 4 depicts the results from real-time free-breathing acquisitions compared to the Cartesian reference cine with segmented breath-hold acquisition, both for a healthy volunteer with regular heartbeat and a patient suffering from arrhythmia. In the breath-hold acquisitions, high image quality was achieved using the xSDNet model. For the healthy participant, both the reference cine and real-time approach produced sharp images. For the patient with irregular cardiac cycles, the ECG-gated cine showed pronounced motion artifacts, while real-time frames reconstructed by xSDNet appeared sharp, with sufficient SNR and low aliasing artifact level. The findings are further supported by dynamic views provided in the supplement for each participant from Fig. 4 (supplementary_video_2.gif and supplementary_video_3.gif). For the healthy participant, slight motion artifacts are visible in the blood pool. For the patient, some instability in segmentation performance appears as temporal jitter around the myocardial tissue and more pronounced in the RV.

### 3.2 Expert reader study

**Fig. 5:**
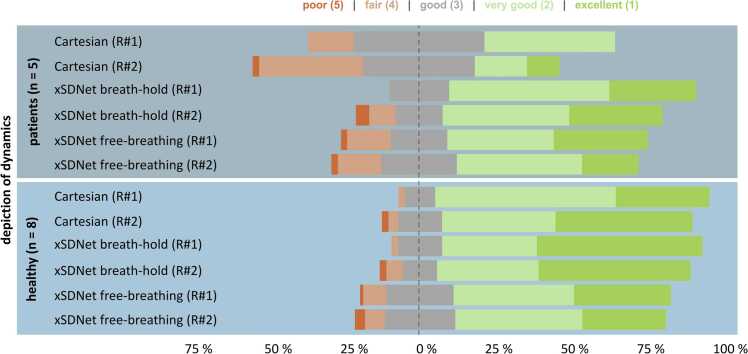
Results from the expert reader study. Distribution of ratings using a 5-point Likert scale (1 = excellent to 5 = poor) are shown separately for both expert readers (R#1 and R#2) for the depiction of dynamics for xSDNet in breath-hold and free-breathing and Cartesian reference. While differences in the methods were marginal in healthy participants (n = 8), a benefit for depiction of dynamics can be seen in patients with arrhythmia (n = 5). *xSDNet* extended spatial decomposition network

In healthy participants, xSDNet in breath-hold and Cartesian cine achieved good ratings for overall image quality (xSDNet breath-hold: 1.99 ± 0.98; Cartesian: 1.94 ± 0.86; p = 0.052) with a slightly decreased impression in xSDNet free-breathing acquisition (xSDNet free-breathing: 2.40 ± 0.98, p < 0.001). In contrast, in patients with arrhythmia, both xSDNet acquisitions yielded better magnitude image quality compared to the reference (xSDNet breath-hold: 2.10 ± 1.28, p < 0.001, xSDNet free-breathing: 2.40 ± 1.13, p < 0.01, Cartesian: 2.68 ± 1.13). When focusing on the depiction of the dynamics of the heartbeat, real-time imaging with xSDNet has no pronounced benefit in healthy participants without arrhythmia (xSDNet breath-hold: 1.70 ± 0.91, free-breathing: 2.13 ± 0.99, Cartesian: 1.91 ± 0.84, Friedman’s p = 0.09). In patients with arrhythmia however, xSDNet can maintain the high quality of dynamics (xSDNet breath-hold 1.87 ± 1.04, p < 0.001, xSDNet free-breathing: 2.22 ± 1.06, p < 0.001), yielding a relevant benefit compared to Cartesian imaging (2.69 ± 1.09, Fig. 5). Additional image ratings are shown in Supplementary Table 1. Intra-observer reliability was good (ICC = 0.77, 95% confidence interval [0.75, 0.79], p < 0.001).

The structural similarity index, the peak signal-to-noise ratio, and the normalized root mean square error were calculated for additional quantitative insights into image quality and are provided in the Supplementary Material under Section 6.

### 3.3 Cardiac function

**Fig. 6:**
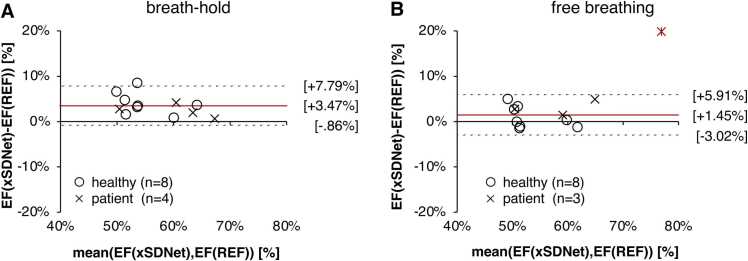
Bland-Altman plots for the ejection fractions determined by different methods. xSDNet represents the automatic segmentations of real-time frames as second output of the proposed neural network in breath-hold (A) and free-breathing (B). REF depicts the manual segmentations of the ECG-gated Cartesian reference. Bias is indicated in brackets and marked as a red line. Upper and lower levels of agreements are indicated and marked as dotted lines. In (B), one patient was additionally excluded from bias and agreement levels due to strong banding artifacts (outlier marked as red cross). *xSDNet* extended spatial decomposition network, *ECG* electrocardiography.

One patient was excluded from functional analysis due to severe banding artifacts in apical slices throughout all acquisition methods which led to poor manual and automatic segmentation. Overall, ejection fractions were widely comparable between methods for healthy participants and arrhythmic patients (Fig. 6). Ejection fractions were slightly higher in breath-hold xSDNet real-time compared to Cartesian reference (bias +3.47%, limits of agreement (LoA) [−0.86, 7.79%], healthy: bias +4.02%, LoA [−0.93, 8.96%], patients: bias +2.37%, LoA [−0.15, 4.89%]. A smaller bias was observed in free-breathing (bias +1.45, LoA [−3.02, 5.91%], healthy: bias +0.86%, LoA [−3.87, 5.59%]; patients: bias +3.01%, LoA [0.22, 5.81%]). However, due to the exclusion of one additional subject due to bindings, the data for the patient group in free-breathing are sparse (Fig. 6, outlier is marked in red).

In the Supplementary Material, segmentation quality was additionally quantified by determining Dice-Sørensen coefficients.

### 3.4 Speed of reconstruction

The prototype implementation of the reconstruction and segmentation pipeline processed images step by step (offline, and on separate workstations) and was not optimized for a clinical workflow. The iterations of CG-SENSE (# 10) for determining an interim estimate for one real-time frame took 2.10 s on an Nvidia Titan RTX GPU (NVidia, Santa Clara, California). Each 2D frame was then subjected to the xSDNet to determine high-quality images and segmentation masks, which took 0.02 s on an Nvidia Titan X.

In addition, coil sensitivities were determined once for each real-time series of a specific slice. Gridding a temporal average on an Nvidia Titan RTX took 0.33 s, while the subsequent application of the algorithm by Walsh et al. [22], which adaptively determines coil maps was still CPU-based and took approximately 3.5 s.

For a typical number of 80 real-time frames, the mean reconstruction time per frame can therefore be estimated at ∼2.2 s.

## 4 Discussion

We proposed xSDNet, a DRL model designed for the joint reconstruction and segmentation of undersampled spiral bSSFP motion studies of the heart. This model enables full coverage of the LV within 1–2 min under free-breathing conditions, substantially reducing scan time compared to segmented breath-hold acquisition that typically requires 10 min or more. In conjunction with the simultaneously delivered segmentation masks, xSDNet has the potential to accelerate acquisition and postprocessing of essential cardiac cine MRI, making it a promising candidate for integration into clinical workflows.

### 4.1 Image quality

In our exploratory feasibility study, xSDNet reconstructions achieved very good to excellent image quality ratings. Image quality was equivalent in real-time breath-hold imaging and Cartesian cine, while ratings were slightly inferior in free-breathing imaging. Conversely, in patients with cardiac arrhythmia, both xSDNet acquisitions in breath-hold and free-breathing showed superior image quality in comparison with Cartesian segmented reference cine MRI (e.g., Fig. 5). The latter exploits binning of data acquired across several heartbeats, which results in excellent image quality for regular cardiac cycles and proper breath-holds. In patients who cannot hold breath or suffer from arrhythmia, this method, however, often delivers insufficient image quality and—maybe more severely—can corrupt derived functional parameters in a subtle manner, when the incorrect assignment of different cardiac phases leads to temporal blurring. In its current implementation, the temporal footprint of real-time frames is 48 ms and images are reconstructed individually, i.e., no temporal model is applied. This excludes any kind of temporal blurring for the images reconstructed by xSDNet, which is confirmed by the ratings of the item “depiction of dynamics.” Solely breathing motion within the temporal window of 48 ms could lead to additional corruption of the data, which should, however, be largely negligible.

In general, the image quality of the real-time series reconstructed by xSDNet in terms of residual artifacts, sharpness, etc., was not inferior to the conventional fully sampled Cartesian cine acquisition. The initial application of 10 conjugate gradient steps exploiting the physical information on coil sensitivities has a positive impact here and stabilizes the overall performance. Residual blurring due to off-resonance can be recognized in the accelerated images in the chest wall. If necessary, however, this can be further reduced using appropriate methods [5], [30], [31].

Nevertheless, it is conceivable to also exploit temporal redundancies within image reconstruction (and segmentation), with potential positive impact on the quality of real-time series (and masks). The advantages of processing frames individually, i.e., the insensitivity to temporal overfitting, would, however, need to be waived with respect to the current implementation of xSDNet.

### 4.2 Assessment of cardiac function

In terms of image segmentation, results for the masks of the blood pool were sufficiently stable to detect local minima and maxima fully automatically, except for a small number of slices, where banding artifacts disturbed the semantic segmentation process of xSDNet. Derived ejection fractions were in good agreement with those from the clinical reference standard, with a slight positive bias for xSDNet at low variances of the deviations. For myocardial tissue and RV, the segmentations were somewhat more unstable, with a higher jitter in temporal observation and more frequent misclassification (see example shown in supplementary_video_3.gif and Dice-Sørensen coefficients in Supplementary Material Section 5). As the determination of myocardial mass or right ventricular ejection fraction from these would have required at least some kind of manual interaction, such as the selection of accurately segmented frames across all real-time frames, we decided not to perform and include this non-fully automated evaluation.

### 4.3 Latency of image reconstruction and segmentation

The current implementation allows a reconstruction time per frame of about 2.2 s on average, which is dominated by the CG-SENSE iterations applied as a pre-step. However, it should be noted that the algorithm was not optimized for overall runtime efficiency yet in this exploratory feasibility study. The following steps can be used to further reduce latency:

Even though estimating coil sensitivities has to be performed only once for a series, the runtime of more than 3 s of the current CPU implementation is still too long. We currently use images with full matrix size (512 × 512) as input for the algorithm proposed by Walsh et al. [22]. As coil sensitivities are smooth, however, this resolution is by far not necessary. Reducing the resolution by only a factor of 4 accelerates the total time for the estimation below 0.25 s.

As no temporal model is applied throughout the series, the CG-SENSE reconstruction of each frame can be run in parallel on a single or multiple GPUs. With a negligible runtime of the neural network modules (0.02 s per frame), this can reduce inference times well below 10 s for a real-time series covering a few RR intervals (2D + *t*), also without high-end hardware.

### 4.4 Comparison with existing methods

Recently, an alternative approach was proposed to reconstruct real-time cardiac MR data and automatically determine left ventricular EF [32]. In contrast to xSDNet, which exploits an integrated procedure to reconstruct and segment data in a multi-tasking fashion, the authors use a two-step approach, which first determines real-time images with a 3D U-Net-based network and then performs the segmentation with another 2D U-Net.

In [15], real-time acquisitions based on spiral sampling were directly reconstructed using a modified fast convolutional neural network architecture, initially proposed for video denoising. For the focus of interventional studies, this allowed a latency of only 33 ms per frame. To adjust xSDNet toward a comparable fast application, the initial CG-SENSE step could be waived. Here, however, we aimed at uncompromised image quality, which would have to be downgraded to some degree (e.g., lower spatial or temporal resolution, residual aliasing artifacts), if the physical information on coil sensitivities remains unconsidered and data are directly fed into the reconstruction and segmentation model.

In [33], a CS model was used to provide real-time cardiac imaging in free-breathing. Acquisitions across multiple heartbeats were registered by a non-rigid correction of respiratory motion to allow for signal averaging and to improve slice positioning. In contrast to this approach, our intention was to not mix information from different RR cycles. To enable a direct comparison between gated and binned data from Cartesian cine MRI and our proposed method, we determined the median of left ventricular volumes across multiple cardiac cycles captured during real-time acquisition.

## 5 Limitations

At 1.5T, the spiral bSSFP pulse sequence, applied with a fixed phase between subsequent excitations of 180° and combined with a local shim as provided by the vendor of the MR system, resulted in hardly any banding artifacts inside the heart throughout all exams to acquire training data. For a small number of single slices of the test data, however, signal cancellations in myocardial tissue and blood pools were obtained, which especially had a major impact on the segmentation performance, both for manual and automatic processing. As xSDNet was applied offline so far, bandings could not be detected immediately after running the pulse sequence. An initial frequency scout would be reasonable to facilitate clinical transfer.

A high number of simulated training sets (set 1) was added in each epoch, mainly to provide a greater variety of cardiac anatomies for the segmentation part of the pipeline. Still, the number of training sets with 100% authentic contrast (set 2) was comparably small, which was most likely the main reason for decreased segmentation performance, especially in apical slices for myocardium and RV (see e.g., Dice-Sørensen coefficients in Supplementary Material Section 5). So far, we delivered initial proof of feasibility based on a comparison with manual masks from Cartesian series only. We are confident that increasing the number of spiral cardiac MR datasets will further stabilize segmentation performance. In general, the initial findings of our exploratory feasibility study need to be further analyzed in a subsequent more extensive study with higher statistical power to rigorously demonstrate its clinical value, including direct comparisons with other state-of-the-art segmentation methods.

The VarNet, we used for comparison, was trained with the spiral bSSFP sets acquired for training purposes only. In this case, the addition of simulated raw data based on the Kaggle set did not improve the scalar metrics reported in Supplementary Material.

Finally, in some cases, the chest wall appears very bright in comparison to the Cartesian reference. This is mainly caused by an image normalization filter, which was automatically applied for the reference Digital Imaging and Communications in Medicines reconstructed by the scanner. For the reconstructions of spiral data, we did not apply such a filter.

## 5 Conclusion

xSDNet applied to undersampled spiral bSSFP acquisitions in free-breathing enables real-time cardiac cine MRI with an image quality comparable to fully sampled Cartesian scans, an automatic determination of ejection fractions in agreement with the clinical reference standard, and significantly shorter scan times of 1–2 min for the entire heart.

## Funding

This work was supported by the German Ministry for Education and Research under Research Grants 05M20WKA and 01KD2215A, and the Interdisciplinary Center for Clinical Research Würzburg under Research Grant F-437.

## Author contributions

Nordbeck Peter: Writing—review and editing, Conceptualization. Kleineisel Jonas: Writing—review and editing, Software. Petri Nils: Writing—review and editing, Validation, Formal analysis. Heidenreich Julius Frederik: Writing—review and editing, Visualization, Supervision, Project administration, Investigation, Formal analysis, Data curation, Conceptualization. Bley Thorsten Alexander: Writing—review and editing, Supervision, Resources, Conceptualization. Baeßler Bettina: Writing—review and editing, Validation, Investigation, Funding acquisition, Conceptualization. Schad Oliver: Visualization, Software, Methodology. Sauer Simon: Formal analysis, Data curation. Wech Tobias: Writing—review and editing, Writing—original draft, Visualization, Validation, Supervision, Software, Project administration, Methodology, Investigation, Funding acquisition, Formal analysis, Data curation.

## Declaration of competing interests

The authors declare the following financial interests/personal relationships which may be considered as potential competing interests: All relationships are outside the current work reported here: Bettina Baeßler declares the following financial interests: CEO & Founder of LernRad GmbH. Speaker Bureau Bayer Vital GmbH. Thorsten A. Bley declares the following financial interests: Director of the Institute for Diagnostic and Interventional Radiology in Würzburg. Consultation and speaking fees for BioTel Research, Bracco, Chugai, Guerbet, Roche, Novartis, Siemens Healthineers. Editor of RöFo: Fortschritte auf dem Gebiet der Röntgenstrahlen und bildgebenden Verfahren. P. Nordbeck has served as speaker/consultant for Abbott, Abiomed, Amicus, Bayer, Biotronik, Boehringer Ingelheim, Boston Scientific, Cardiac Dimensions, Daiichi, Idorsia, Medtronic, Sanofi, Takeda.

## Data Availability

The source code of the presented xSDNet model is publically available at https://github.com/expRad/xSDNet/.
