# Supplementary material for: Joint image reconstruction and segmentation of real-time cardiovascular magnetic resonance imaging in free-breathing using a model based on disentangled representation learning
Source: J Cardiovasc Magn Reson. 2025 Jan 24;27(1):101844. doi: 10.1016/j.jocmr.2025.101844 (PMC11874730; doi:10.1016/j.jocmr.2025.101844)
Supplement: Supplementary file 1 — Supplementary material [file mmc1.pdf]

Supplementary Material

Joint image reconstruction and segmentation of real-time cardiac MRI in free-breathing using a model based on disentangled representation learning

Tobias Wech, Oliver Schad, Simon Sauer, Jonas Kleineisel, Nils Petri, Peter Nordbeck, Thorsten A. Bley, Bettina Baeßler, Bernhard Petritsch, Julius F. Heidenreich

1. MR pulse sequence

The spiral balanced steady state free precession pulse sequence as illustrated in Fig. 1 of the main document is applied both in breath-hold and free breathing. The acquisitions in free breathing were solely used for real-time-depictions, which represents the intended and proposed application.

The acquisitions performed using a breath-hold were both used for further validation of real-time imaging <u>and</u> to obtain training data in healthy volunteers. For the latter, pairs of corresponding images, i.e. corresponding undersampled real-time and fully sampled "label" images, were required for the supervised training scheme applied. This was made possible, by a dedicated way of rotating the pattern shown in Fig. 1B of the main document along the temporal dimension (see Supp. Fig. 1):

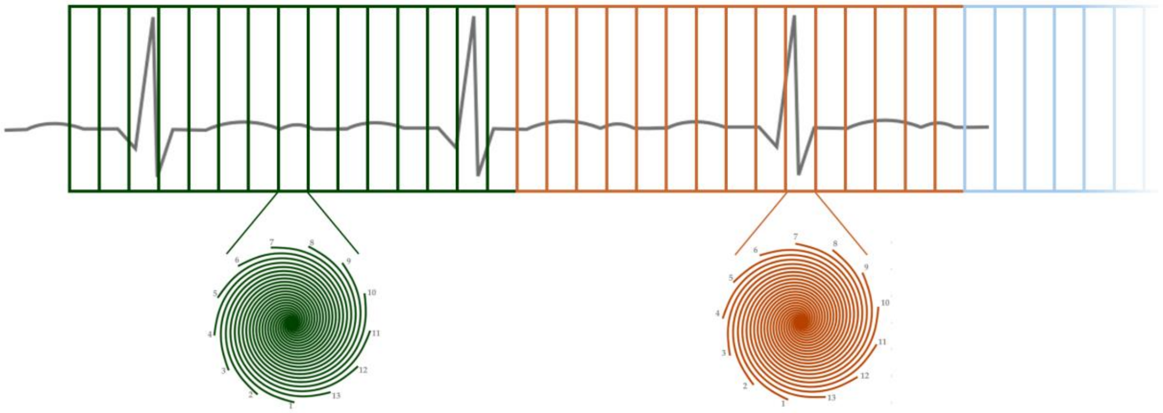

Supplementary Figure 1: Rotation of sampling pattern after a number of frames n.

The trajectory of one real-time frame consisting of 13 equally distributed spiral arms (green pattern in Supp. Fig. 1, corresponding to a temporal footprint of 48 ms) was played out repeatedly for a number of n frames which are in sum exceeding the length of one RR interval (i.e. $n \cdot 48\ ms > RR$). Subsequently the pattern was rotated to fill the largest gaps (orange pattern) and was then again applied for the same number of n frames. This was repeated 8 times, while each new pattern filled the largest gaps of the trajectory obtained

by cumulative combination of all preceding patterns. Combining all 8 pattern ultimately delivers a trajectory which is “fully sampled”.

By chosing $n \cdot 48\, ms > RR,$ each cardiac phase (with a temporal accuracy of $\sim$ 48 ms) is sampled with each of the 8 patterns at least once, and as the acquisition was performed in breath-hold, the procedure then allows to determine fully sampled “segmented” frames and corresponding undersampled real-time frames for network training. n thus had to be chosen individually for the current heart rate of the volunteers scanned for training.

When acquiring and reconstructing data in real-time only, there is no more need for building fully sampled frames in a segmented fashion for each heart-phase. In order to be able to determine temporal average images, however, we still applied the same pattern, but used considerably smaller values for n (typically $n = 10$). As the start of the measurements is not triggered, the acquisition of $8 \cdot n$ (in our case ~80) ensures the coverage of at least one RR cycle (in our case 3-4 cycles).

2. xSDNet architecture

The xSDNet architecture (see Fig. 2 in main document) is derived from the SDNet architecture introduced by Chartsias et al. [1]. The latter disentangles cardiac MRI images into spatial anatomical factors, which are capturing the physical structure of the heart etc., and non-spatial modality factors, which reflect how the image was acquired, and are thus independent from the actual anatomy of the individual patient (i.e. imaging modality, (MRI-) protocol, contrast agent, …). The proposed original SDNet architecture was designed to analyze MR images in that respect, and thus to learn the two separate factors simultaneously. This comes with potential advantages of improved analysis of anatomical features, potentially leading to more accurate assessment of heart function and the ability to combine information from various imaging modalities (e.g., MRI with CT scan) by focusing on the common anatomical factors.

The authors highlight the general nature of the approach to factorize data into intuitive, meaningful and interpretable components, which inspired us to extend the concept to reconstruct and segment undersampled MR data, and thus to accelerate MRI. The ability of the method to derive binary anatomy maps (see Fig. 3a in [1]) was conserved in our case, even though undersampling / streaking artefacts are now superimposed on the input images. In particular, the maps could not only split up modality information, but also the artefacts originating from violating the Nyquist criterion (see $\bar{a}$ in the center of Figure 2, which represents maps of an inference of the model to patient data), and are

thus comparable to those shown in [1]. The anatomy maps were used to derive segmentation maps by an additional shallow network.

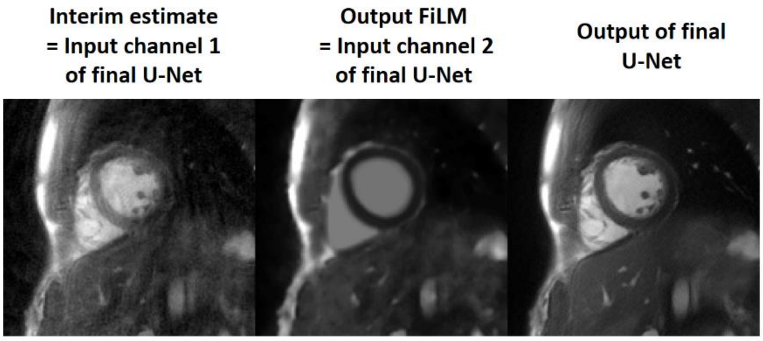

Supplementary Figure 2: The two input channels and the output channel of the final U-Net in Fig. 2 of the main manuscript (illustrated in green) for an inference on test data.

Both the anatomy maps and the input image are used to derive the modality latent space by a variational autoencoder (identical architecture as in [1]), which is encoding information on contrast, thereby representing the “image modality”. The two latent spaces (anatomy and modality) are then recombined by the “FiLM” module to obtain a reconstruction, as in the original SDNet. The obtained images – both in the original publication (see e.g. Fig. 1 and 3 in [1]) and also for our data (see Output FiLM in Supp. Fig. 2) – again represent both anatomical and modality features, however also appear somewhat “cartoonish” or oversimplified. However, for our application on accelerated scans, this estimated reconstruction also no longer features the undersampling artefacts, which are superimposed on the original image. We therefore used both the interim estimate and the output of the FiLM layer as inputs for the final U-Net, to deliver images free from artefacts but still containing detailed anatomical information (see image on the right in Supp. Fig. 2).
To study the benefit of this two-channel-approach, a comparison of the reconstruction performance of the xSDNet architecture with a radically ablated version, using the final U-Net only (trained with identical data, but just using the interim image as single input), was already presented in our ISMRM abstract [2]. There, a reader study confirmed, that xSDNet outperforms the benchmark U-Net for all acceleration factors applied (see Table 1 of the abstract and Fig. 3 for a visual impression).

3. Results: Detailed image ratings from the expert reader study

Additionally to overall quality of the magnitude images and the depiction of dynamics along the temporal domain, subjective image rating were performed to assess artefacts in bloodpool and myocardium as well as sharpness of the endocardial and epicardial contours.

	all (n=13)			patients (n=5)			healthy participants (n=8)		
	Cartesian	xSDNet BH	xSDNet FB	Cartesian	xSDNet BH	xSDNet FB	Cartesian	xSDNet BH	xSDNet FB
Artifacts in the bloodpool	1.91±0.88	1.62±0,88	1.84±0.92	2.12±1.06	1.52±1.06	1.92±1.10	1.79±0.76	1.65±0.80	1.79±0.81
Artifacts in the myocardium	2.22±0.96	1.91±0.95	2.32±1.03	2.52±1.14	1.93±1.14	2.53±1.15	2.06±0.82	1.83±0.96	2.21±0.95
Sharpness endocardium	2.05±1.06	1.93±1.08	2.42±1.14	2.46±1.12	2.29±1.22	2.60±1.20	1.82±0.97	1.98±1.03	2.32±1.10
Sharpness epicardium	2.03±1.00	2.13±1.14	2.47±1.12	2.37±1.12	2.02±1.15	2.68±1.17	1.85±0.89	1.86±1.02	2.34±1.04
Temporal dynamics	2.12±1.02	1.76±0.98	2.39±1.07	2.69±1.09	1.87±1.04	2.22±1.06	1.91±0.84	1.70±0.91	2.13±0.99
Magnitude image quality	2.02±1.03	2.07±1.10	2.17±0.69	2.68±1.13	2.10±1.28	2.40±1.13	1.94±0.86	1.99±0.98	2.40±0.98

Supplementary Table 1: Data are shown as mean ± standard deviation for patients (n=5), healthy participants (n=8) and pooled (n=13). BH = breathhold, FB = free breathing (5-point Likert scale: 1 = excellent to 5 = poor).

4. Results: Real-time acquisitions in free breathing – comparison of image reconstruction based on xSDNet and Variational Network

An additional expert reader study compared the image quality of xSDNet reconstruction in free breathing with an alternative reconstruction approach with VARNET. As expected, xSDNet and VARNET reconstructions achieve similar ratings on the sharpness of endocardial/epicardial contours and the depiction of dynamics. With regards to artefacts in bloodpool and myocardium ratings were favourable for the xSDNet approach.

	patients (n5)		
	Cartesian	xSDNet FB	VARNET FB
Artifacts in the bloodpool	2.12±1.06	1.92±1.10	2.16±0.71
Artifacts in the myocardium	2.52±1.14	2.53±1.15	2.96±0.81
Sharpness endocardium	2.46±1.12	2.60±1.20	2.62±1.00
Sharpness epicardium	2.37±1.12	2.68±1.17	2.67±1.08
Temporal dynamics	2.69±1.09	2.22±1.06	2.23±0.94
Magnitude image quality	2.68±1.13	2.40±1.13	2.46±0.86

Supplementary Table 2: Data are shown as mean ± standard deviation for patients n=5 in free breathing (5-point Likert scale: 1 = excellent to 5 = poor).

5. Results: Dice-Sørensen coefficient

In a subset of the test dataset comprising breath-held spiral acquisitions in five healthy volunteers with regular heartbeat, we were able to determine fully sampled segmented cine data with frames matching the real-time reconstructions by xSDNet (same procedure as performed for obtaining training data). The frames resulting from reconstructing the fully sampled (spiral) data were manually segmented for left ventricle, right ventricle and myocardium by the same expert as above. These masks were then

used to calculate the Dice-Sørensen coefficient with respect to the masks resulting from xSDNet for the corresponding real-time frames.

The generation of this additional reference (fully sampled spiral) was necessary, as the Cartesian reference cine series are not adequate here, due to a different rotation angle and spatial resolution with respect to the spiral acquisitions. It is well known that small segments (especially slices at the apex) can disproportionally impact the Dice-Sørensen coefficient in a negative way. Therefore we both calculated median with inter quartile (iqr) range as well as mean with standard deviation (std) to summarize the obtained values for all slices evaluated.

**DICE**	Left Ventricle	Right Ventricle	Myocardium
median ± iqr	0.94 ± 0.05	0.85 ± 0.16	0.84 ± 0.10
mean ± std	0.91 ± 0.12	0.78 ± 0.18	0.80 ± 0.16

For the left ventricle both central tendency and spread of the Dice-Sørensen coefficient are good, allowing robust derivation of e.g. ejection fraction. Corresponding measures for right ventricle and myocardium are lower, indicating the need for additional training data for theses compartments to reach the required robustness.

6. Results: Quantitative assessment of image quality using scalar metrics

By providing matching frames of fully sampled spiral (segmented acquisition) and corresponding real-time frames, as reconstructed by xSDNet and alternative methods, the data generated in 5. can be used to calculate scalar metrics like the structural similarity index (SSIM), the peak signal-to-noise ratio (PSNR) as well as the normalized root mean square error (NRMSE). Values are given as mean ± std across all slices.

**Scalar metric**	LRS	VarNet	xSDNet
SSIM	0.89 ± 0.028	0.92 ± 0.028	0.92 ± 0.024
PSNR [dB]	32 ± 2.7	34 ± 3.2	33 ± 2.3
NRMSE	0.12 ± 0.035	0.094 ± 0.033	0.11 ± 0.019

The scores for xSDNet are overall comparable to those achieved by the Variational Network. With respect to the low rank plus sparse model, slightly higher SSIM values could be obtained.

References for supplement

[1] Chartsias A, Joyce T, Papanastasiou G, Williams M, Newby D, Dharmakumar R, Tsaftaris SA. Disentangled Representation Learning in Cardiac Image Analysis. Med Image Anal 2019;58:101535. https://doi.org/10.1016/j.media.2019.101535.

[2] Wech T, Heidenreich J, Bley T, Baessler B. A disentangled representation trained for joint reconstruction and segmentation of radially undersampled cardiac MRI. Annu. Meet. ISMRM, 2022, p. 16.
