# Supplementary figures and images for: Joint image reconstruction and segmentation of real-time cardiovascular magnetic resonance imaging in free-breathing using a model based on disentangled representation learning
Source: J Cardiovasc Magn Reson. 2025 Jan 24;27(1):101844. doi: 10.1016/j.jocmr.2025.101844 (PMC11874730; doi:10.1016/j.jocmr.2025.101844)

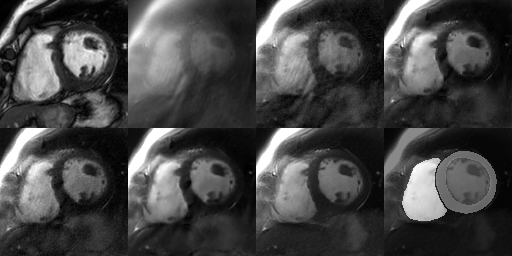

Supplement: Supplementary file 2 — Supplementary material [file mmc2.zip › supplementary_video_1.gif]

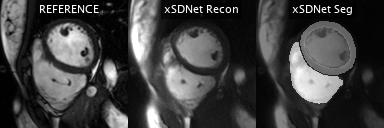

Supplement: Supplementary file 3 — Supplementary material [file mmc3.zip › supplementary_video_2.gif]

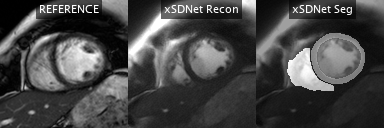

Supplement: Supplementary file 4 — Supplementary material [file mmc4.zip › supplementary_video_3.gif]
